# General principles governing the amount of neuroanatomical overlap between languages in bilinguals

**DOI:** 10.1016/j.neubiorev.2021.08.005

**Published:** 2021-08-13

**Authors:** Monika M. Połczyńska, Susan Y. Bookheimer

**Affiliations:** Department of Psychiatry and Biobehavioral Sciences, David Geffen School of Medicine at UCLA, University of California, Los Angeles, CA, USA

**Keywords:** Age, Bilingual, Brain surgery, Clinical language mapping, Cognitive control, fMRI, Model, Multilingual, Proficiency, Principles, Effort

## Abstract

The literature has identified many important factors affecting the extent to which languages in bilinguals rely on the same neural populations in the specific brain region. The factors include the age of acquisition of the second language (L2), proficiency level of the first language (L1) and L2, and the amount of language exposure, among others. What is lacking is a set of global principles that explain how the many factors relate to the degree to which languages overlap neuroanatomically in bilinguals. We are offering a set of such principles that together account for the numerous sources of data that have been examined individually but not collectively: (1) the principle of acquisition similarity between L1 and L2, (2) the principle of linguistic similarity between L1 and L2, and (3) the principle of cognitive control and effort. Referencing the broad characteristics of language organization in bilinguals, as presented by the principles, can provide a roadmap for future clinical and basic science research.

## 1. Introduction

There has been a tremendous interest in how languages are organized in individuals who are bilingual, i.e., people who know more than one dialect or language (Fabbro, 2013). Based on neuroimaging studies with healthy volunteers, lesion studies with patients suffering from post-stroke aphasia, and neurosurgical reports using invasive mapping techniques, we know that languages in bilinguals can neuroanatomically overlap or they can be represented separately (Fernández-Coello et al., 2017; Hernandez et al., 2000; Klein et al., 2002; Paradis, 1977; Perani et al., 1998; Sulpizio et al., 2020). By neuroanatomical overlap, we recognize areas that are specific to the first (L1) and the second (L2) language (a co-localization of regions subserving L1 and L2 function). On the other hand, by neuroanatomical separation, we understand regions that are spatially different. In studies using electrocorticography, convergence and divergence are operationally based on whether a single site that is stimulated disrupts one language versus two languages, or neither. Generally, electrical stimulation covers a spatial extent of about half a centimeter, which would suggest that neuroanatomical convergence or divergence is at least within a five mm spatial extent. In most fMRI studies, contrasts of tasks performed in L1 and L2 are applied (L1 > L2 and L2 > L1). In cases where there were no differences between the contrasts, a complete overlap between L1 and L2 is assumed. On the other hand, if either of the contrasts produced specific brain regions, these were interpreted as areas of separation. These contrasts have a lower resolution than results obtained with electrocorticography (details to follow below).

There are several ways in which investigators have examined language representation in bilinguals. Early lesion studies on bilinguals focused primarily on the differential loss of one language in bilingual patients (or more severe deficits in one language than the other), and non-parallel recovery patterns (e.g., one language recovering before the other) (Hernandez, 2013; Pitres, 1895a, b). Thanks to the more recent advances in non-invasive (functional magnetic resonance imaging, fMRI) and invasive (Wada testing) techniques, we can look at the extent of bilateral engagement in bilinguals (Hull and Vaid, 2007; Połczyńska et al., 2017). Another way to study language organization in bilingual individuals is to examine the degree to which the same regions overlap for L1 and L2, which can be performed with fMRI but also invasively using electrocorticography (Emmorey et al., 2008; Fernández-Coello et al., 2017; Hirosh and Degani, 2018; Liu and Cao, 2016; Ou et al., 2020; Perani et al., 1998). In a recent systematic review of 28 studies on neurosurgical language mapping in bilingual patients (Połczyńska and Bookheimer, 2020), both separate and shared neuroanatomical representation of L1 and L2 was found to be common. Specifically, all the reviewed studies reported at least some regions in the brain that had a divergent organization of L1 and L2, and 82 % of the studies found areas shared by L1 and L2.

The literature started to address the neuroarchitecture of languages in bilingual individuals over 100 years (Pitres, 1895a, b; Fabbro, 1999; Hernandez, 2013). Many different factors have been demonstrated to affect the extent to which languages rely on the same neural populations in a specific brain region (Sulpizio et al., 2020). The factors include the age of acquisition of L2, proficiency level of L1 and L2, the amount of exposure to each language, the manner of L2 acquisition (implicit versus explicit), the linguistic distance (typological similarity) between L1 and L2, and the modality of acquisition of L1 and L2 (oral versus signed) (Emmorey et al., 2008; Hirosh and Degani, 2018; Hull and Vaid, 2007; Liu and Cao, 2016; Ou et al., 2020; Perani et al., 1998). We will refer to these factors as language modifiers (LMs). Numerous studies have examined how each of these LMs influences the neural representation of languages in bilinguals (e.g., Buchweitz and Prat, 2013; Xi et al., 2017). Some of the LMs have been associated with neuroanatomical convergence (e.g., early age of L2 acquisition) (Fernández-Coello et al., 2017), and others have been linked with independent regions of language specialization (e.g., later age of L2 acquisition) (Wartenburger et al., 2003). What is lacking is a set of global principles that explain why and how these factors modify the divergent versus convergent neuroanatomical organization of languages in bilinguals (Cargnelutti et al., 2019).

At least two methodological challenges are responsible for the fact that there have been no guiding principles determining how LMs influence the neural representation of bilingualism. First, most neuroimaging studies (e.g., Kim et al., 2016; Perani et al., 1998) have only focused on one or – less frequently – two LMs at a time (e.g., age of L2 acquisition, and/or L2 proficiency) without controlling for the remaining LMs that also play a role in shaping the organization of languages in the brain (e.g., how often subjects used each of their languages, how linguistically similar or different the examined languages were, whether the subjects learned their L2 formally or informally, or whether they knew any other foreign languages). Investigating only selected LMs without considering all the remaining LMs may result in a high amount of variability and data bias. Second, there has been considerable heterogeneity in subject samples across studies. Some reports have compared mono- versus bilinguals (e.g., Hull and Vaid, 2007). Other studies have examined bilingual groups that differed with regards to a selected LM or two (Perani et al., 1998).

Here we propose a set of organizing principles that predict the amount of neuroanatomical convergence between L1 and L2 in the brain. The principles build on a large body of literature that encompasses previous results on the numerous LMs and bilingual cognitive control. The general principles proposed in the current work complement prior important models of neurocognitive adaptations in the bilingual brain (e.g., Pliatsikas, 2020; DeLuca et al., 2020; Abutalebi and Green, 2008) by offering predictions on how LMs modulate the amount of neuroanatomical convergence in bilinguals, and cognitive adaptations. Wherever possible, we included both studies involving healthy volunteers and clinical studies that applied invasive methods such as direct cortical stimulation. Each technique has its unique mapping characteristics. Specifically, direct cortical stimulation is only used to map language functions for surgical purposes (Lucas et al., 2004). It has a high spatial resolution in comparison to neuroimaging (less than 5 mm versus the practical spatial resolution of 7–8 mm in fMRI, following acquisition smoothing and spatial normalization). Thus, referencing studies using invasive techniques can provide more precise data on the neural representation of languages in bilinguals and further our understanding of the principles that govern the amount of neuroanatomical co-localization between L1 and L2. Moreover, direct cortical stimulation is specific in showing areas that are essential for language, whereas fMRI displays regions that may be engaged in language, even if they only support language function and are not primary language sites (Połczyńska et al., 2015; Rofes et al., 2015).

The general principles proposed here provide a foundation for a theoretical understanding of how languages are organized in bilinguals. They also have practical significance in helping clinicians make decisions about how to map languages in surgical patients.

## 2. Methods

We conducted a systematic literature search using PubMed. The search was performed between November 2018 and June 2020. We closely followed the methods of our recently published review (Połczyńska and Bookheimer, 2020). However, that study was limited to bilingual research using clinical language mapping. The reviewed reports in our prior work used electrocorticography (applied in the majority of the studies), pre-operative fMRI, and the Wada test. Our previous review examined how six LMs modulate the amount of neural overlap between L1 and L2. Of these six LMs, we obtained conclusive results for three: (1) the age of L2 acquisition, (2) proficiency of L2, and (3) the linguistic distance between L1 and L2. The present study is a significant expansion of our 2020 work. Here, we searched the literature to determine how eight LMs impact the degree of neuroanatomical co-localization between L1 and L2, and how each of these factors modifies cognitive control function. To supplement the clinical studies included in our 2020 review (n = 29), we conducted an additional, targeted literature search of studies on healthy individuals using 53 keywords (for a complete list of the keywords, see Appendix A). This targeted search added 57 peer-reviewed papers (see Appendix B for a list of the additional studies). Thus, the current review totaled 86 studies. We excluded studies presenting clinical research other than clinical language mapping (e.g., studies on bilingual aphasia after stroke). We also excluded papers that were literature reviews and studies that were not published in English.

## 3. General organizing principles explaining how LMs modulate neuroanatomical localization of L1 and L2 in bilinguals

**Fig. 1.**
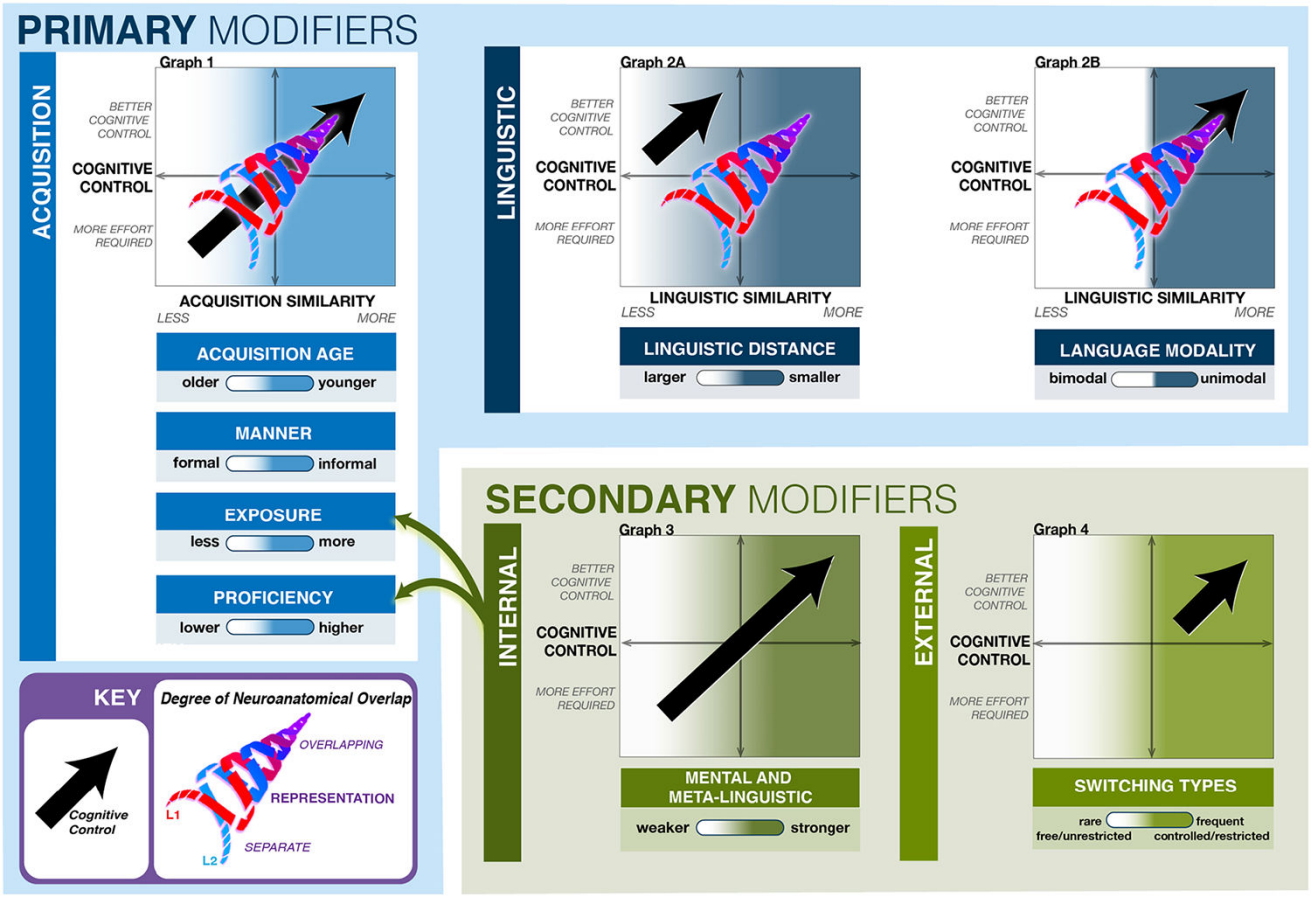
Principles governing the neuroanatomical localization of languages in bilingual individuals. The swirl represents the degree of neuroanatomical co-localization of L1 and L2, whereas the black arrow stands for cognitive control (see the Key box in the bottom left corner). The primary modifiers can modulate both the amount of neuroanatomical overlap between L1 and L2, and cognitive control. In Graph 1, the more acquisition similarity between L1 and L2, the more neuroanatomical convergence between the languages. Specifically, L1 and L2 are more likely to co-localize if: acquisition age was young for both languages; both languages were learned informally, both are used on a daily basis, and both are spoken with a high degree of proficiency. Conversely, L1 and L2 are more likely to be organized separately if: L1 was acquired at a young age and L2 was learned at an older age, L1 was acquired informally and L2 was learned formally, there is a high amount of exposure to one language and a low amount of exposure to the other language, and one language is highly proficient and the other one has low proficiency. Acquisition similarity is colinear with cognitive control in Graph 1 – the more acquisition similarity, the better cognitive control; the less acquisition similarity, the more cognitive effort is required. Graphs 2A and 2B show that the more linguistic similarity between L1 and L2, the more neural convergence can be anticipated: a smaller linguistic distance between L1 and L2 (Graph 2A), and L1 and L2 share the same modality – signed or spoken (i.e., they are unimodal; Graph 2B). On the other hand, the less linguistic similarity, the more divergence is expected: a larger linguistic distance between L1 and L2 in Graph 2A, and L1 and L2 having a different modality (i.e., they are bimodal) in Graph 2B. The linguistic modifiers affect cognitive control selectively: better cognitive control is predicted by a larger linguistic distance (the top left square of Graph 2A), and shared language modality (unimodal; the top right square of Graph 2B). The secondary modifiers indirectly impact the amount of neuroanatomical overlap through modulating two acquisition modifiers: exposure and proficiency (dark green arrows). The secondary modifiers can affect cognitive control. In Graph 3, the stronger the mental and meta-linguistic abilities, the better cognitive control; the weaker the mental and meta-linguistic abilities, the more effort is required. In Graph 4 (the top right square), frequent and controlled/restricted language switching is associated with better cognitive control.

Here, we propose the classification of LMs as being primary or secondary, along with their subtypes. The primary LMs are linguistic factors that modulate the amount of neuroanatomical convergence between L1 and L2. They can also impact cognitive control. The secondary LMs are non-linguistic factors that can indirectly modify the degree of neuroanatomical overlap between L1 and L2 by affecting selected primary LMs. The secondary LMs also modulate cognitive control (see Fig. 1). We also offer a set of three principles that organize the impact of LMs on the amount of neuroanatomical convergence between L1 and L2, and cognitive control. The first two principles explain and define the extent of neuroanatomical overlap between L1 and L2 based on specific LMs. The first principle concerns the similarity in how each of the languages was acquired. The second principle is based on the similarity between languages themselves. The third principle explains how the degree of neural language convergence between two languages modulates cognitive control.

### 3.1. The primary modifiers

The literature has demonstrated several factors known to directly modulate the amount of neuroanatomical convergence between L1 and L2 (Emmorey et al., 2008; Hirosh and Degani, 2018; Hull and Vaid, 2007; Liu and Cao, 2016; Ou et al., 2020; Perani et al., 1998). We will refer to these LMs as the primary modifiers, and we will further divide them into two groups:

- *Acquisition modifiers* – relate to the way languages are acquired. They include the age of language acquisition (younger versus older), proficiency level (higher versus lower), the amount of language exposure (higher/balanced versus lower/imbalanced), and the manner of L2 acquisition (formal versus informal),
- *Linguistic modifiers* – the properties of the languages themselves. They comprise the linguistic distance (smaller versus larger) and language modality (unimodal versus bimodal). Recently, we presented a simple way to measure the linguistic distance between two languages by counting how many branches apart the two languages are on a language family tree (Połczyńska and Bookheimer, 2020). The linguistic distance can be classified into: (1) very close – languages sharing similar lexicon, grammar, and phonetic-phonological systems with a high amount of intelligibility. The languages are one branch apart on a language family tree (e.g., Swiss German, and German), (2) close – languages that are relatively similar. They are two branches apart on a language tree (e.g., German and Danish), (3) moderate – languages that are three branches apart on a language tree (e.g., German and Italian), and (4) distant – languages that are four or five branches apart (e.g., German and Farsi, and German and Tongan, respectively. Distant languages are highly unintelligible to each other (Połczyńska and Bookheimer, 2020). As for language modality, bilinguals who speak two oral languages are referred to as “unimodal” because they use the same modality (oral) to communicate in each of their languages. A person who is hearing impaired and who communicates in two different sign languages will also be referred to as a unimodal bilingual. On the other hand, we call an individual who speaks a sign and an oral language a bimodal bilingual because they can communicate via two different modalities (oral and signed).

The two types of the primary language modifiers are presented schematically in Fig. 1. The acquisition and linguistic modifiers are governed by two common principles that determine whether L1 and L2 are more likely to converge or have a distinct neuroanatomical representation.

#### 3.1.1. The principle of acquisition similarity between L1 and L2

The first and most robust organizing principle we offer predicts that the similarity between the language modifiers in L1 and L2 predicts the amount of neuroanatomical co-localization. The more similar each of the acquisition modifiers is between L1 and L2, the more neuroanatomical co-localization is expected between the languages. In Fig. 1, the principle is illustrated with a “swill” inside Graph 1. The swirl is made up of two woven lines that show a divergent representation of L1 (red) and L2 (blue) (also see the Key box in the bottom left corner of Fig. 1). The two lines combine to form a purple line representing a convergent organization of L1 and L2. A higher degree of neural overlap is more likely to occur when both L1 and L2 were acquired early in life, they were both learned informally, they are used on a daily basis, and they are both spoken with a high degree of proficiency. The less similar acquisition between L1 and L2, the greater the neuroanatomical separation. Examples of differences between LMs in L1 and L2 include: early acquisition of L1 versus late learning of L2, informal acquisition of L1 versus formal learning of L2, a high amount of exposure to one language and a low amount of exposure to the other language, and high L1 proficiency versus low L2 proficiency, (see Fig. 1).

A body of literature on healthy volunteers and clinical studies applying invasive techniques both support the principle of acquisition similarity. In the remaining paragraphs of this section, we will discuss one acquisition modifier per paragraph. Each paragraph will include background research on a given modifier, including neuroimaging studies in healthy volunteers and clinical reports that applied invasive techniques.

For the age of acquisition, most studies that have compared the representation of the cerebral language network in early (simultaneous) versus late (sequential) bilinguals have used ages three to six as a common cutoff to delineate between early and late language learning (Berens et al., 2013; Hull and Vaid, 2007; Kovelman et al., 2009). Note, however, that various language domains have different optimal acquisition windows (J. Paradis, 2004; M. Paradis, 2009; Ullman, 2005). For example, native-like pronunciation in L2 is more likely to be achieved if L2 was acquired during the first years of life. On the other hand, a new lexicon can still be learned at a high proficiency level by older children or even adults (Werker and Hensch, 2015a, b). There is fMRI evidence indicating that, neuroanatomically, early healthy bilinguals have their languages represented more bilaterally than monolinguals and late bilinguals (Hull and Vaid, 2007). The same has been reported for bilingual neurosurgical patients using fMRI (Połczyńska et al., 2017). Among those individuals, the amount of L1 activity in the left hemisphere appears to be the same in bilingual patients and their monolingual patient controls. There are also no differences in the amount of activity between L1 and L2 in the left hemisphere between bilingual patients and their controls. However, compared to monolinguals, bilinguals display increased activity in the right hemisphere for both L1 and L2, which characterizes a more bilateral language representation (Połczyńska et al., 2017). Within-hemisphere localization has been examined using studies performing invasive language mapping and research that applied non-invasive neuroimaging in healthy bilinguals. Most neuroimaging reports on healthy bilinguals that have examined the differences between early and late healthy bilinguals have shown more neuroanatomical overlap in the former group and more neuroanatomical separation in the latter group (e.g., Fernández-Coello et al., 2017; Kim et al., 1997; Połczyńska et al., 2016; Wartenburger et al., 2003). While a few scattered studies on healthy bilinguals (e.g., Ou et al., 2020) reported the opposite, i.e., more neuroanatomical divergence between L1 and L2 in early compared to late bilinguals, we have not found data in any studies using invasive mapping methods that would confirm this opposite finding.

The manner of language acquisition can be “informal/implicit” or "formal/explicit". Informal language learning involves an implicit, naturalistic language setting outside a classroom. An example of informal language learning would be a child acquiring two different languages from their caretakers at home (the caretakers may or may not be native speakers of these languages). An informal acquisition can also occur when an older individual moves to another country and learns a new language mainly by interacting with speakers of the new language. On the other hand, formal language learning typically takes place in a school or a language course setting. An individual learning L2 in an explicit way is taught how to speak, comprehend, read, and write in that language. We are not aware of any neuroimaging research on the impact of the manner of acquisition of natural languages on the amount of neuroanatomical convergence between L1 and L2 in the brain. However, distinct functional networks have been posited to subserve implicit versus explicit learning of artificial grammar using fMRI (Seger et al., 2000). The electrophysiological organization of artificial grammar learned implicitly versus explicitly (as measured with event-related potentials) showed a similar electrophysiological organization to native speakers for implicitly learned artificial grammar (Morgan-Short et al., 2012). This finding might indicate that when L1 and L2 were both acquired implicitly, they are more likely to have a shared cerebral representation.

The amount of language exposure refers to the proportion of language use in L1 and L2. It can be more substantial for L1 than L2 or balanced between L1 and L2. It is also not uncommon to see a more considerable amount of exposure to L2 than to L1. One example of greater L2 exposure is that of highly proficient bilinguals who have resided in a country of their L2 for a long time (particularly if they moved there earlier in life). One challenge with the research on the amount of language exposure in bilinguals has been substantial variations in how data for this LM was collected. The variations include estimates of daily, weekly, or an overall distribution of L1 and L2 use (DeAnda et al., 2016). For the purposes of this work, the amount of language exposure was classified as higher (daily, frequent/occasional), and lower (rare and none). The amount of language exposure appears to have a role in modulating the degree of neuroanatomical convergence between L1 and L2, which was confirmed by early lesion studies (Pitres, 1895a, b; see Abutalebi and Green, 2008 for a discussion). Certain aspects of L2 (e.g., the articulatory system) may be more plastic to exposure, while other aspects (e.g., orthography) may be less influenced by this LM (Meschyan and Hernandez, 2006). We assume more neuroanatomical convergence in bilinguals with a balanced amount of exposure to their languages, and more separation in bilinguals with unbalanced exposure to their languages. In cases where the amount of exposure to one of the languages is low, areas associated with cognitive effort can be observed, particularly when proficiency in that language is lower (Meschyan and Hernandez, 2006; Wartenburger et al., 2003). In individuals with a higher amount of exposure, a certain amount of separation can still be expected. In our recent review (Połczyńska and Bookheimer, 2020), all the included studies (n = 29) reported a divergent localization of L1 and L2, in addition to regions of co-localization. Most bilingual patients in the reviewed research (87 %) used their languages daily. For instance, a study that performed extraoperative cortical stimulation found that L1 and L2 were organized as a combination of co-localizing and divergent sites in a teenage boy. The boy suffered from intractable seizures following a temporal anaplastic astrocytoma in the left hemisphere. He had been exposed to his L1 (English) and L2 (Hebrew) daily since birth and was highly proficient in both languages. Following his surgery, the boy experienced deficits in Hebrew but not English (Serafini et al., 2008).

One common classification of L2 proficiency is based on the Common European Framework of Reference for Languages (“Common European Framework of Reference for Languages (CEFR),” 2019). The classification applies to L2 production, comprehension, writing and reading: (a) low/beginner (CEFR levels A1 and A2) – the learner has minimal communicative abilities, (b) intermediate (CEFR levels B1 and B2) – using connected text, the speaker can take part in a conversation on more basic topics, and (c) high/advanced (CEFR levels C1 and C2) – the speaker has very high L2 fluency and is able to interact with native speakers naturally. Similar to the age of L2 acquisition, L2 proficiency is a significant variable affecting the organization of languages in the brain (Perani et al., 1998). Healthy bilinguals with low L2 proficiency are more likely to have less considerable neuroanatomical co-localization between their languages (e.g., Chee et al., 2001; Leonard et al., 2011; Perani et al., 1998; Wartenburger et al., 2003; Yetkin et al., 1996). Clinical studies using invasive language mapping have not replicated this finding (e.g., Lucas et al., 2004). However, most of the clinical studies included highly proficient L2 speakers with no or few low L2 proficiency speakers (Roux et al., 2004; Walker et al., 2004). Languages with a low proficiency level are typically not mapped for neurosurgical purposes.

Taken together, the principle of acquisition similarity predicts and is consistent with the literature showing that when L1 and L2 are acquired similarly, they are more likely to have an overlapping neuroanatomical representation.

#### 3.1.2. The principle of linguistic similarity between L1 and L2

The principle of linguistic similarity explains the neuroanatomical representation of L1 and L2 in bilinguals based on the two linguistic modifiers (the linguistic distance and modality). When the linguistic modifiers are similar for L1 and L2, there is a higher likelihood that the two languages will have an overlapping neural organization (see the upper part of the swirl in Graph 2A, Fig. 1). On the contrary, if the linguistic modifiers are considerably different for L1 and L2, the languages are more likely to be organized more divergently in the brain (see the lower part of the swirl in Graph 2A, Fig. 1).

While the linguistic distance between languages is probably a less robust LM than, for example, the age of language acquisition, it may still modulate the cerebral organization of languages to a certain extent. Structural characteristics of languages (e.g., orthography) have been indicated to modify the neurocognitive architecture (e.g., learning to read and write), particularly in bilinguals who were exposed to their L2 during the first years of life (before age five) (Jasińska et al., 2017). Electrical stimulation language mapping has demonstrated that the more distant L1 is from L2, the more separate neural organization may be expected (Lucas et al., 2004). The literature on healthy bilinguals has shown that there may be more L1 and L2 divergent sites when languages are distant (Jeong et al., 2007). For instance, in addition to shared neuroanatomical areas, reading alphabetic languages (e.g., English) versus logographic languages (e.g., Chinese) recruits divergent brain regions. One example is a more significant engagement of the right fusiform gyrus and inferior occipital lobe during reading in Chinese than English (Bolger et al., 2005; Mei et al., 2015; Tan et al., 2005). Another study (Jeong et al., 2007) found more similar fMRI activity between Japanese and Korean (linguistically closer) than between English and Korean (linguistically more distant) in native Korean trilinguals. As evidenced by studies on healthy individuals and neurosurgical patients (Kim et al., 2016; Kochunov et al., 2003), typologically similar languages are more likely to have a convergent neuroanatomical distribution. Concurrently, neurosurgical studies using invasive mapping techniques demonstrated that those languages may still show some amount of neuroanatomical separation. For example, one study using intraoperative brain stimulation on a patient with a brain tumor demonstrated that two linguistically very close languages (German and Swiss German) had several areas of neuroanatomical divergence, in addition to co-localizing sites (Połczyńska et al., 2016). One possible explanation might be that a certain degree of neuroanatomical divergence has a facilitating role in the suppression of language interference (i.e., being able to monitor one language when speaking the other; Połczyńska and Bookheimer, 2020; Kroll et al., 2015). One possibility is that having some extent of separate neural representations for similar linguistic aspects in L1 and L2 helps compartmentalize each language as separate independent microanatomical networks operating within partially overlapping brain sites (Borius et al., 2012; Xu et al., 2017). Future studies using resting-state fMRI could elucidate our understanding of the impact of linguistic distance on language suppression.

The modality of language acquisition can be spoken or signed. One of the differences between a spoken and sign language is that they do not share a sub-lexical level, which excludes cross-language activations, i.e., co-activation caused by sounds or letters shared by two spoken languages (Giezen and Emmorey, 2016). Compared with non-signer monolinguals, both L1 and L2 signers tend to have more robust right hemisphere activity. Signing is a unique spatial language skill that unsurprisingly engages the right hemisphere (Bavelier et al., 1998; Williams et al., 2016). Bimodal bilinguals have less significant neuroanatomical overlap than unimodal bilinguals (see the bottom part of the swirl in Graph 2B, Fig. 1). Robust differences between bi- and unimodal bilinguals have been shown both during language production (e.g., more activation in the left posterior middle temporal and bilateral parietal regions when signing versus speaking; Soderfeldt et al., 1997) and comprehension (the bilateral auditory cortex is activated more robustly during oral than sign comprehension; Zou et al., 2012).

In sum, consistent with the prior research, the principle of linguistic similarity explains that when L1 and L2 are linguistically similar, their neural representation is more likely to co-localize.

### 3.2. The secondary modifiers

We introduce the term “secondary modifiers” to describe non-linguistic factors that can impact L2 attainment (Benjamin et al., 2018; Ibrahim, 2020; Masgoret and Gardner, 2003; Michel et al., 2019; Nicol and De France, 2020). We propose the division of indirect modifiers into those that are internal and those that are external. The modifiers are presented in Fig. 1. The internal modifiers include (but may not be limited to) factors such as metalinguistic awareness, motivation, attitude, personality, aptitude, prejudice, emotions (i.e., positive L2 enjoyment), memory, and attention (Ibrahim, 2020; Masgoret and Gardner, 2003; Michel et al., 2019; Nicol and De France, 2020; Dziubalska-Kołaczyk and Wrembel, in press). We will call these modifiers mental and metalinguistic modifiers. The external modifiers include two types of language switching modifiers: (1) the frequency of language switching (frequent versus rare) (Prior and Gollan, 2011; Verreyt et al., 2016) and (2) the type of language switching (controlled/restricted versus free/unrestricted) in the bilingual’s daily life (de Bruin, 2019; Green and Abutalebi, 2013). The internal modifiers are governed by a principle we describe below.

#### 3.2.1. The effect of the secondary internal modifiers

As demonstrated in Fig. 1, the secondary modifiers do not modulate the degree of neuroanatomical convergence directly (see the lack of the swirl in Graphs 3 and 4 in Fig. 1). Yet, the internal modifiers indirectly modulate the amount of neuroanatomical overlap between L1 and L2 because they may affect L2 attainment. In particular, they can influence: (1) proficiency (e.g., good attention and memory abilities can lead to higher L2 proficiency, but when these abilities are poor, L2 proficiency could be lower; see Michel et al., 2019), (2) the amount of exposure (e.g., a negative attitude toward L2 could result in a smaller amount of time that an individual is willing to devote to learn L2) (Ibrahim, 2020; Masgoret and Gardner, 2003; Michel et al., 2019; Nicol and De France, 2020). The impact of the internal modifiers on the two primary modifiers is depicted in Fig. 1, with the dark green arrows running from the internal modifiers toward proficiency and exposure.

### 3.3. The principle of cognitive control and effort

Since bilinguals have more than one language system, they need to decide which language to speak in given circumstances. Languages are assumed to be jointly active and speakers need to constantly focus on the language they want to communicate in (Kroll et al., 2015; Abutalebi and Green, 2008; Bialystok et al., 2012). Based on studies involving healthy individuals, we know that the constant language selection engages a left fronto-temporo-parietal network that is, however, not language-specific. Typically reported regions include the basal ganglia (the caudate), anterior cingulate cortex, inferior frontal gyrus, middle frontal gyrus, dorsolateral prefrontal cortex, superior temporal sulcus, and inferior parietal lobule (Borius et al., 2012; Calabria et al., 2018; Hernandez et al., 2001, 2000; Kho et al., 2007; Moritz-Gasser and Duffau, 2009; Price et al., 1999; Sierpowska et al., 2018, 2013; Wang et al., 2007, 2013). The network has been shown to be active when bilinguals speak each of their languages (Abutalebi et al., 2013; Abutalebi and Green, 2008; Calabria et al., 2018; Jones et al., 2012; Petitto and Kovelman, 2003). Henceforth, we will refer to “language selection” as “language switching”. However, by “switching” we do not understand switching one language off while the other language is switched on (switching on and off was implicated by earlier studies) involving bilinguals (e.g., Wang et al., 2007). Rather, we refer to switching attention from one language to the other while both languages remian active (Bialystok et al., 2012, 2009).

Several studies that performed invasive language mapping reported that the left middle frontal gyrus plays an important role in language switching (Lubrano et al., 2012; Sierpowska et al., 2018, 2013; Wang et al., 2007). The largest clinical language mapping study on language switching to date included nine patients with a brain tumor (Sierpowska et al., 2018). The patients were Spanish-Catalan bilinguals (seven early and two late bilinguals). The authors employed an object naming and a language switching task both during a preoperative fMRI and electrical stimulation mapping. Using the latter mapping method, language switching regions were identified in eight of the nine patients. The authors found a clear predominance of the middle frontal gyrus during language switching over other areas. Moreover, in another study (Sierpowska et al., 2013), the same group reported post-operative impairments in language switching in a patient whose left middle frontal gyrus was removed. Based on their findings, Sierpowska et al. (2018) suggested that the left middle frontal gyrus may be the main mediator or a hub for language switching in bilingual individuals.

The ability to switch between languages while maintaining a dual system mode has been referred to as *bilingual cognitive control* or *cognitive effort* (Cargnelutti et al., 2019; Green and Abutalebi, 2013). While these two terms often seem to be used interchangeably, we are explicitly differentiating them as separate entities here. Bilingual cognitive control has been suggested to be an instantiation of the domain-general executive system. It includes processes such as attention, planning, selection, task switching, inhibition, and monitoring (Calabria et al., 2018). By bilingual cognitive control, we presume better performance on executive function tests generally (van den Noort et al., 2019), cognitive reserve (Bialystok et al., 2014), and more robust activation of the cognitive control network during resting-state fMRI (Berken et al., 2016; Kousaie et al., 2017). As such, bilingual cognitive control can be seen in, for example, more proficient bilinguals with considerable exposure to their languages.

In addition to recruiting regions associated with cognitive control, in some L2 learners (e.g., individuals with low L2 proficiency), we may see brain activity associated with *cognitive effort* (Skulmowski and Rey, 2017). Cognitive effort can be interpreted as a global increase in attention that may not be specific to any brain region. It can be seen as increased computation and activity within the language network itself (Hasegawa et al., 2002). Cognitive effort may also require additional support from areas that are engaged with working memory (Howard et al., 2015). For example, a study that applied pupillometry showed that lower L2 proficiency was correlated with a later pupil response in word retrieval, which is indicative of a higher cognitive effort (Schmidtke, 2014). Cognitive effort is more likely to be observed in bilinguals with less L2 exposure and a lower proficiency level (Meschyan and Hernandez, 2006; Wartenburger et al., 2003). When presenting the principle of cognitive control and effort in the section below, we will re-evaluate the existing findings based on the distinction between bilingual cognitive control and cognitive effort.

Bilingual cognitive control or cognitive effort may not affect language organization, but they will impact the appearance of language representation, as shown in activation studies. Therefore, it is crucial to consider them in evaluating brain images of, for example, individuals who may require extra cognitive effort. These individuals may display additional activity that may be unrelated to language. Such areas of additional activity can easily distort the assessment of language lateralization, for instance. In particular, areas such as the anterior cingulate and the dorsolateral prefrontal cortex can have bilateral representation because of cognitive effort.

The principle of cognitive control and effort predicts associations between the amount of neuroanatomical overlap and cognitive modulations in bilingual individuals. In Fig. 1, cognitive control is represented with a black arrow (see the Key box in the bottom left corner).

#### 3.3.1. Cognitive adaptations and primary modifiers

For the primary acquisition modifiers, the principle predicts that the degree of neuroanatomical overlap is *always colinear* with cognitive control: the more overlap between L1 and L2, the better the cognitive control is expected; the greater separate representation between L1 and L2, the more cognitive effort is required. Hence, the swirl and the black arrow run collinearly in Graph 1 (Fig. 1). In parallel with similar acquisition modifiers where there is a higher probability of greater neuroanatomical overlap between L1 and L2, superior cognitive control is more likely for: younger age of L2 acquisition, a high proficiency level of L1 and L2, a high and balanced amount of exposure to L1 and L2, and informal learning of L2 (see the top right square in Graph 1, Fig. 1). More cognitive effort is anticipated (along with a greater separate neuroanatomical representation) for an older age of L2 acquisition, a lower proficiency level in L1 or L2, less frequent (imbalanced) exposure to L1 or L2, and explicit learning of L2 (see the bottom left square in Graph 1).

For the age of acquisition L2, both early and late bilinguals have been found to engage brain sites associated with cognitive effort, but early bilinguals activated these regions to a smaller extent (Cargnelutti et al., 2019). Early simultaneous bilinguals also seem to have superior cognitive control when compared with late bilinguals (e.g., suppression of language interference; Kousaie et al., 2017). It is plausible to assume that explicit L2 learning may require more cognitive load, particularly in early learning stages, compared to early implicit L2 acquisition. A study by Linck et al. (2008) provided mixed results. Among other groups, the authors compared learners of Spanish immersed in a natural, Spanish-speaking environment with individuals who were learning Spanish in a classroom. The classroom learners performed significantly better on a Simon task compared to the immersed learners. Yet, the authors conducted another experiment, and the finding was not replicated there (Linck et al., 2008). Next, for the amount of language exposure, resting-state fMRI has shown that simultaneous bilinguals (L1 and L2 acquired since birth) displayed stronger functional connectivity within the bilingual cognitive control network at rest than sequential (L2 learned later in life) bilinguals (Berken et al., 2016; Kousaie et al., 2017). Finally, proficiency can also modulate bilingual cognitive control – it reduces the cognitive load in highly proficient bilinguals (Cargnelutti et al., 2019) and it is associated with a greater mean N2 amplitude (greater inhibition) on executive testing (Fernandez et al., 2013). Bilinguals with lower proficiency in one of their languages may recruit non-classical language areas, including, for instance, brain regions associated with articulatory effort (Chee et al., 2001; Perani et al., 1998; Yetkin et al., 1996).

In contrast to the primary acquisition modifiers, the effect of overlapping representation is not perfectly colinear for the primary linguistic modifiers. A smaller linguistic distance between L1 and L2 does not appear to be associated with more cognitive effort. For this reason, in Fig. 1, there is no black arrow in the bottom right part of Graph 2A. However, when the linguistic distance is more substantial, we may expect both a greater neuroanatomical separation and better cognitive control (see the black arrow in the top right square of Graph 2A). Antoniou et al. (2016) investigated executive control in three groups – children speaking two languages (bilingual), children speaking two dialects (bilectal), and monolingual children. The authors observed that the bilingual children had superior performance on executive control tasks compared with the bilectal children, and the bilectal children performed better on the tasks than the monolingual children (Antoniou et al., 2016). Hence, speaking two languages that are typologically different may increase the probability of better performance of executive function tasks than speaking typologically very close languages. As to language modality, unimodal bilinguals are more likely to have excellent cognitive abilities and more neuroanatomical co-localization than bimodal bilinguals (Emmorey et al., 2008) (see the black arrow in the top right square of Graph 2B). This is likely because bimodal bilinguals can use the so-called code-blends, i.e., simultaneously produced oral words (e.g., English) and signs (e.g., American Sign language). Code-blends are assumed to have a computationally lower cost than using only one language while suppressing production in the other language (Emmorey et al., 2008). At the same time, we have found no evidence suggesting that unimodal bilingualism itself is associated with elevated cognitive effort (Olulade et al., 2016), which can also be noted via the absence of a black arrow in the bottom left square of Graph 2B).

#### 3.3.2. Cognitive adaptations and secondary modifiers

It is predicted that the stronger the secondary internal modifiers, the better the cognitive abilities. When these LMs are weaker, more cognitive effort may be anticipated (see the black arrow in Graph 3, Fig. 1). The impact of the secondary internal modifiers on cognitive adaptations is estimated to be less pronounced compared to the magnitude of the impact of the primary acquisition modifiers. Note that we have not found any studies examining the influence of individual modifiers on bilingual cognitive control in the context of L2 learning.

According to the principle of cognitive control and effort, the external secondary modifiers (the frequency and type of language switching) can selectively predict better cognitive abilities. This is illustrated with a black arrow in the top right square of Graph 4, Fig. 1. For the frequency of language switching, one study (Prior and Gollan, 2011) reported that Spanish-English bilinguals who switched between their L1 and L2 frequently in daily life displayed smaller switching costs in a non-verbal task than monolinguals. Conversely, bilinguals (Chinese-English) who rarely switched between their languages daily performed the same as the monolinguals. In line with these results, another study (Verreyt et al., 2016) found that frequent language switchers outperformed rare language switchers on tasks assessing inhibitory control, for instance, the Simon task and the flanker task. Concurrently, we do not assume that rare language switching is strongly linked to cognitive effort (no arrow in the left bottom square of Graph 4, Fig. 1). For controlled/restricted language switching between L1 and L2 (i.e., when L1 and L2 are usually used separately), we presume an ongoing control of the unused language and better performance on tasks requiring inhibitory control. On the other hand, free/unrestricted language switching may not involve a switching cost, and therefore, it may require less cognitive control. As in rare language switching, there seems to be no evidence to imply that free/unrestricted language switching requires additional cognitive effort.

Taken together, the principle of cognitive control and effort predicts associations between the amount of neuroanatomical overlap and cognitive modulations in bilingual individuals through the primary and secondary modifiers.

## 4. Discussion

We proposed three broad principles that account for the many variables predicting the amount of neuroanatomical overlap between L1 and L2. First, the more *similar* the way in which two languages were acquired, the more neuroanatomical co-localization is expected between them. The less similar the way in which L1 and L2 were acquired, the greater the neuroanatomical separation. Second, the similarity between the languages themselves also predicts the amount of neuroanatomical overlap between languages. Third, the amount of neuroanatomical co-localization between L1 and L2 can modulate cognitive control and cognitive effort. While cognitive control does not affect language organization per se, it can impact the appearance of language representation, as shown in activation studies. The effect of the amount of convergent representation is always colinear with cognitive control for the primary acquisition modifiers (the greater the neuroanatomical convergence, the better the cognitive abilities; the smaller neuroanatomical the convergence, the more cognitive effort is required). The impact of the degree of overlapping neuroanatomical organization is not perfectly colinear with cognitive control for the primary linguistic modifiers. The secondary external modifiers are not affected by the amount of co-localizing neural organization, but they can selectively predict better cognitive abilities. The three primary principles can account for the multiple factors studied in the literature that have been separately shown to predict the degree of overlap between L1 and L2 in the brain.

The architecture of different languages in the brain began to raise scientific questions when early clinical reports showed selective deficits in only one language in bilingual aphasics (Hernandez, 2013; Paradis, 1977). Recovery from aphasia in bilingual individuals may be parallel (simultaneous recovery of two languages, similar severity of language deficits) or non-parallel (one language recovers before the other) (Paradis, 1977). Parallel recovery takes place in 40 % of bilingual patients, and non-parallel recovery constitutes the rest. Among cases with non-parallel recovery, in about a half of patients, L1 improves better than their L2, and for the other half of patients, the opposite has been reported (Fabbro, 1999). Toward the end of the 19th century, neuropsychologists determined factors underlying the distribution of L1 and L2 in the brain. The main factors included: (1) the age of acquisition – L1 recovers first (Ribot’s law), and (2) the amount of exposure – the most dominantly used language recovers first (Pitres’ law) (Hernandez, 2013; Pitres, 1895a, b). To add further complexity, selective aphasia in bilinguals has also been reported in specific language aspects within a single language, e.g., reading with or without impaired writing (Ku et al., 1996; Luria, 1960), language comprehension (Silverberg and Gordon, 1979), and production (Winslow, 1868). Based on these findings, languages were assumed to have at least partially separate representation in the brain (Obler and Albert, 1978; Scoresby-Jackson, 1867). At the same time, some authors have disagreed, claiming that all languages are localized in overlapping regions (Fabbro, 1999; Paradis, 2001; Pitres, 1895a, b).

Thanks to imaging techniques and invasive clinical language mapping methods (e.g., electrocorticography), we have gained further understanding of how various LMs modulate the neural organization of L1 and L2. The literature has demonstrated that bilingualism is associated with intricate structural and functional adaptations in the brain that also affect cognitive performance (Vaughn et al., 2020; Cargnelutti et al., 2019; Fernández-Coello et al., 2017; Abutalebi et al., 2013; Abutalebi and Green, 2008). The adaptations occur in response to a wide range of experience-based factors. There is a growing understanding that the effects of bilingualism on the function and structure of the brain and cognitive performance are complex. Unveiling these effects is complicated by individual differences among bilinguals in their language experience, as well as the characteristics of the languages themselves (DeLuca et al., 2020; Połczyńska and Bookheimer, 2020; Dziubalska--Kołaczyk and Wrembel, in press; de Bruin, 2019). A number of models have helped integrate the existing findings. For example, the Dynamic Restructuring Model makes predictions about structural adaptations (decreases and increases in the integrity of the grey and white matter) in three phases of increasing bilingual experience (initial exposure, consolidation, and peak efficiency) (Pliatsikas, 2020). Another recent Unifying the Bilingual Experience Trajectories model provides a set of comprehensive predictions about neurocognitive adaptations based on four experienced-based factors, including the duration of bilingual experience, proficiency, language switching, and diversity and intensity of language use (DeLuca et al., 2020). The general principles proposed in the current work complement the prior models by offering predictions on how LMs modulate neuroanatomical convergence in bilinguals and cognitive adaptations.

All the principles offered in this work account for the fact that each bilingual is different because of the complex interplay among the multiple LMs. Because of the individual differences among bilinguals, it remains challenging to generalize over larger samples (de Bruin, 2019; Dziubalska-Kołaczyk and Wrembel, in press). Dziubalska-Kołaczyk and Wrembel (in press) discuss these challenges in the context of multilingual speakers. They use the term *situation of acquisition* to embrace different variables in every multilingual case in each of their languages (L1, L2, or L3+), such as proficiency level, a formal/informal learning context, and individual modifiers.

An important question to consider is how the interactions between various LMs modulate the degree of neural overlap between L1 and L2. The age of acquisition appears to be the most potent factor determining language representation in the bilingual brain (Fernández-Coello et al., 2017; Połczyńska et al., 2016; Walker et al., 2004). We speculate that, when accounting for all the other modifiers, the age of language acquisition is an overriding factor. The robustness of this LM in modulating the degree of neuroanatomical convergence between two languages may be linked to early experience-dependent changes in the structure of the brain. During the first two years of life, there is a tremendous loss of neurons. The loss will be less significant if the neuropathways are involved in L2 exposure and use. For example, infants can discriminate between speech sounds of all languages. Within the first few months of life, the auditory system starts to tune into the sounds of the language (or languages) of its environment, with a concurrent loss of the ability to discriminate speech sounds that are not experienced (Werker and Hensch, 2015a, b). Thus, neurons that are active together in response to the sounds of the languages from the infant’s environment (e.g., Spanish and English), increase their connectivity. On the contrary, neurons which are not coincidentally active will have weaker connections (Kolb et al., 2017). Due to synaptic pruning, the weak connections will eventually disappear, and the child will no longer be able to discriminate the speech sounds which they have not been exposed to (e.g., speech sounds from languages other than English and Spanish; Kolb et al., 2017; Bogacka et al., 2006). Hence, the early exposure to L2 requires additional neural resources and in the developmental trajectory results in long-term changes, and less cortical pruning of synapses in the language system that normally occurs early in life. Neuroplastic changes after the optimal periods have closed are still possible throughout the lifespan. However, they differ quantitatively and qualitatively from the mechanisms that are biologically programmed to take place within the optimal periods (Berken et al., 2016). Thus, from a neurobiological perspective, the increased functional overlap in early bilinguals appears plausible.

While it has been demonstrated that LMs other than the age of L2 acquisition can modify the amount of neuroanatomical co-localization (Emmorey et al., 2008; Hirosh and Degani, 2018; Hull and Vaid, 2007; Liu and Cao, 2016; Ou et al., 2020; Perani et al., 1998), their impact is suggested to be secondary. Concurrently, while the impact may be less robust, its magnitude appears to vary for different LMs. For example, one study (Giroud et al., 2020) showed that it was the early age of L2 acquisition and not the amount of exposure (50 % versus 100 %) that positively impacted behavioral and brainstem plasticity in a study comparing early English-French bilinguals. The bilingual adults were compared to two groups of late bilingual adults (English-French and French-English) (Giroud et al., 2020). The results demonstrated that the early English-French group performed as well as the late English-French (L1 = English) group identifying category boundaries in English vowels. The late French-English bilinguals (L1 = French) performed worse than the early English-French bilinguals (Giroud et al., 2020). Other LMs may modulate the amount of neuronal co-localization between L1 and L2 in early bilinguals more substantially than the amount of exposure. For example, Kovelman et al. (2014) reported differences in brain activations between early hearing bimodal bilinguals (English and American Sign Language) and two groups of unimodal monolinguals (deaf signers using American Sign Language and English monolinguals). Using functional near-infrared spectroscopy, the authors found that the bimodal bilinguals displayed decreased activation in the left parietal areas while signing, relative to the deaf signers.

We speculate that in late bilinguals, in whom more neuroanatomical divergence is expected, other acquisition LMs can further modulate the degree of neuroanatomical convergence more substantially than in early bilinguals. For instance, late and highly proficient bilinguals are more likely to have more considerable neuroanatomical overlap than late bilinguals with low L2 proficiency (Chee et al., 2001; Krefta et al., 2015). Late bilingual individuals with a balanced daily exposure to both of their languages are predicted to have more neural convergence when compared to late bilinguals with unbalanced daily exposure to their languages (e.g., they use their L1 daily but L2 rarely). The manner of language acquisition is always informal for early bilinguals, while late bilinguals are more likely to learn their L2 in a formal setting. While there is little research on late bilinguals who acquired their L2 informally, studies using implicit learning of artificial grammar suggest that informal L2 acquisition implies more shared neural representation when compared to explicit grammar learning. Further research is required to advance our understanding of this topic.

While we provide a theoretical approach to predicting neural language overlap in bilinguals with our principles, there are also clear, practical applications of this knowledge. For example, when patients are evaluated for neurosurgical planning, these principles can help guide what additional language mapping techniques might be needed, as well as which functional tasks might be necessary to ensure that patients do not come out of brain surgery with deficits in their L1, L2, or both. Further, we have argued that it is important to consider cognitive control activations when interpreting the results of activation studies in surgical patients. This is especially relevant to activation methods as opposed to stimulation techniques (Brennan et al., 2016). The current problem is that many centers are giving up invasive techniques in favor of non-invasive activation methods. When this is the case, it is essential to consider activations that may come from cognitive control (particularly cognitive effort) in bilingual patients.

While we have focused more generally on language representation in bilinguals, there are other aspects of language organization that have not been examined in the bilingual context and, therefore, cannot be integrated into our principles at this time. For instance, one such aspect is the choice of language tasks that can generate different mapping results (Benjamin et al., 2017; Lee et al., 2008). A diverse range of 67 linguistic and cognitive tasks was applied in the reviewed studies. The most commonly used tasks are found in Table 1, in which the object naming task tops for being used the most frequently. The remaining 47 tasks appeared in single studies, and they are listed in Appendix C. We agree with Cargnelutti et al. (2019) that future studies should examine how various LMs modulate the cerebral representation of different language domains (e.g., syntax versus phonology) in L1 and L2.

The list of LMs incorporated into our principles may not be exhaustive, which may be a possible caveat. For instance, one variable which has not been studied in the context of neuroanatomical overlap between languages in bilinguals is the linguistic context. Many people speak colloquially in L1 without the ability to read. On the other hand, depending on the context, they can speak colloquially but also formally in their L2 (e.g., in a professional environment). The additional formal context can be linguistically richer (e.g., deeper vocabulary with a higher degree of semantic complexity) than the informal (colloquial) context. It is unknown whether and how this factor could modify the neural representation of languages.

Another potential LM might be additional external acquisition factors that could indirectly modulate the cerebral organization of L1 and L2 in the brain. The external factors include (and are not limited to) language teachers/instructors, different L2 learning methods, speakers from the learner’s environment (e.g., native, non-native), etc. Hopefully, future research will provide more insights into the roles that these factors might have in modulating the representation of languages in bilinguals. Finally, we realize that some information that the principles include (e.g., inconclusive results on the manner of L2 acquisition on cognitive control; Linck et al., 2008) need further validation in future research.

While we think it is important to refer to clinical language mapping studies where possible, we acknowledge that the presence of a brain lesion can impact language organization in bilingual patients to some extent. For example, a brain tumor or arteriovenous malformations can reduce language dominance (Gohel et al., 2019; Pouratian et al., 2000; Połczyńska and Bookheimer, 2020). Further, whereas most studies on healthy bilinguals have used fMRI, most clinical language mapping studies have performed invasive techniques (most commonly, awake surgery), sometimes in combination with fMRI (e.g., Walker et al., 2004). It should be noted that each language mapping technique has its idiosyncrasies that may influence language mapping results (Benjamin et al., 2017; Hernandez and Li, 2007; Połczyńska et al., 2016). For instance, fMRI can incorrectly identify certain areas as eloquent, whereas in fact, these regions only support language function. In order to verify which areas shown on fMRI as active are critical for intact language function, invasive techniques such as awake surgery are performed (Benjamin et al., 2017; Połczyńska et al., 2015)

## 5. Conclusions

We have offered a set of global principles that predict how numerous language modifiers modulate the neuroanatomical representation of language in bilingual individuals and cognitive adaptations. The principles encompass results from neuroimaging studies on healthy bilinguals and neurosurgical language mapping reports involving bilingual patients. While the principles provide a theoretical approach to predicting neural language overlap in bilinguals, there are also clear practical clinical applications of this knowledge. In patients who are evaluated for neurosurgical planning, these principles can help guide what additional language mapping techniques might be needed, as well as which functional tasks might be necessary to ensure that patients do not come out of brain surgery with deficits in their L1, L2, or both. In cases when non-invasive activation methods are used to evaluate language lateralization and/or representation in bilingual surgical patients, it is essential to consider activations that can come from cognitive control. These activations may be unrelated to language organization as such, particularly in individuals with lower L2 proficiency who require extra cognitive effort. Referencing the broad characteristics of language organization in bilinguals, as presented in the set of principles, may provide a roadmap for future basic and clinical science research.

## Supplementary Material

Appnedix

Appendix

## Figures and Tables

**Table 1:** The most commonly used linguistic and cognitive tasks in the reviewed research. Since many studies examining cognitive control in healthy individuals applied behavioral tests, neuroimaging and behavioral tasks are presented separately. A few (but not all studies) studies administered the same task(s) both with neuroimaging and behavioral methods. Behavioral tasks assessing language proficiency were not included.

Domain	Task category	Task	Healthy controls neuroimaging	behavioral	Clinical mapping: ESM, fMRI, Wada	All reviewed studies
Linguistic	Semantic	Object naming	13	6	24	43
		Auditory responsive naming	0	0	3	3
		Verbal responsive naming	0	0	2	2
		Pyramids and Palm trees	0	2	0	2
		Verbal fluency	2	2	1	5
		Word generation	1	0	0	1
		Verb generation	1	0	2	3
		Peabody picture vocabulary test	0	3	0	3
		Action naming	0	0	2	2
	Grammar/syntax	Sentence syntactic violation task	3	2	0	5
	Comprehension	Sentence comprehension	2	0	1	3
	Sentence-level listening	Story listening	2	0	0	2
	Reading	Word reading	5	0	3	8
		Sentence reading	2	0	5	7
		Implicit reading	0	0	2	2
	Sequential recitation	Counting	0	0	5	5
Cognitive	Switching	Object naming with language switching	9	4	3	16
		The Color-Shape task	1	2	0	3
	Attention, inhibition	Flanker task	1	2	0	3
	Intelligence	The WAIS matrix reasoning test	0	2	0	2

## Appendix

### Appendix A. Keywords used in the literature search

The following keywords (and their combinations) were used to search for the literature on bilinguals: “bilingual*”, “multilingual*”, “language mapping”, “L2”, “second language”, “foreign language”, “fMRI”, “functional magnetic resonance imaging”, “TMS”, “transcranial magnetic stimulation”, “MEG”, “magnetoencephalography”, “PET”, “positron emission tomography”, “EEG”, “electroencephalography”, “direct electrical stimulation”, “awake surgery,” “electrocorticography”, “Wada test”, “intracarotid amobarbital procedure”, “clinical”, “epilepsy”, “brain tumor”, “arteriovenous malformation”, “AVM”, “age of acquisition”, “proficiency”, “exposure”, “manner”, “formal”, “informal”, “implicit”, “explicit”, “linguistic distance”, “linguistic similarity”, “language similarity”, “modality” “bimodal”, “sign language”, “cognitive effort”, “cognitive advantage”, “cognitive abilities”, “executive function”, “language switching”, “metalinguistic awareness”, “motivation”, “attitude”, “aptitude”, “attention” “memory”, “personality”, and “prejudice”.

### Appendix C. Tasks applied in single reviewed studies

Studies with healthy controls:

- sentence listening
- simple declarative sentences
- phoneme categorization
- sentence generation
- retrieval of high and low imaginary words
- visual rhyming judgment
- word repetition
- vowel recognition
- semantic size judgment
- picture selection after story listening
- story watching
- word categorization judgment
- production of syntactic structures
- Ketter String Judgments using artificial grammar,
- Wug test
- word finding vocabulary test
- story reading
- word reading with language switching
- word translation
- backward digit span
- Corsi blocks task, soccer task
- Raven’s colored progressive matrices
- auditory go/no-go task, picture naming with distractor words
- vowel recognition, Attentional Control Scale
- action-object switching
- Simon task
- verbal switching
- nonverbal switching
- Cuttell Culture Fair Intelligence Test
- digit naming, lexical decision
- semantic judgment
- silent narration
- task-switching
- Rapid Naming Test
- Kaufman Brief Intelligence Test: Matrices subtest
- letter-number sequencing.

Clinical language mapping studies:

- famous people naming
- translation from L2 to L1
- L1 reading with L2 responding
- synonym generation
- antonym generation
- sentence completion
- alphabet recitation
- verbal instructions
- a semantic judgment of true-false sentences
- color/shape naming
- repetition
