# Supplementary material for: General principles governing the amount of neuroanatomical overlap between languages in bilinguals
Source: Neurosci Biobehav Rev. Author manuscript; Available in PMC 2022 Mar 28. (PMC8958881; doi:10.1016/j.neubiorev.2021.08.005)
Supplement: Appnedix [file NIHMS1783655-supplement-Appnedix.docx]

**APPENDIX 2**

**Journal articles with primary research studies on healthy controls used in the current work**

Abou-Ghazaleh, A., Khateb, A., Nevat, M., 2020. Language Control in Diglossic and Bilingual Contexts: An Event-Related fMRI Study Using Picture Naming Tasks. Brain Topography, 33(1):60–74. https://doi.org/10.1007/s10548-019-00735-7

Abutalebi, J., Della Rosa, P.A., Ding, G., Weekes, B., Costa, A., Green, D.W., 2013. Language proficiency modulates the engagement of cognitive control areas in multilinguals. Cortex. https://doi.org/10.1016/j.cortex.2012.08.018

Abutalebi, J., Annoni, J.M., Zimine, I., Pegna, A.J., Seghier, M.L., Lee-Jahnke, H., Lezeyras, F., Cappa. S.F., Khateb, A., 2008. Language control and lexical competition in bilinguals: An event-related fMRI study. Cerebral Cortex 18, 1496–1505. https://doi.org/10.1093/cercor/bhm182

Antoniou, K., Grohmann, K.K., Kambanaros, M., Katsos, N., 2016. The effect of childhood bilectalism and multilingualism on executive control. Cognition 149, 18–30. https://doi.org/10.1016/j.cognition.2015.12.002

Bavelier, D., Corina, D., Jezzard, P., Clark, V., Karni, A., Lalwani, A., Rauschecker, J.P., Braun, A., Turner, R., Neville, H.J., 1998. Hemispheric specialization for English and ASL: Left invariance-right variability. Neuroreport 9, 153

Berken, J.A., Chai, X., Chen, J.-K., Gracco, V.L., Klein, D., 2016a. Effects of Early and Late Bilingualism on Resting-State Functional Connectivity. J. Neurosci. 36, 1165–72. https://doi.org/10.1523/JNEUROSCI.1960-15.2016

Bialystok, E., Peets, K.F., Moreno, S., 2014. Producing bilinguals through immersion education: Development of metalinguistic awareness. Appl. Psycholinguist. 35, 177–191. https://doi.org/10.1017/S0142716412000288

Chee, M.W.L., Hon, N., Lee, H.L., Soon, C.S., 2001. Relative language proficiency modulates BOLD signal change when bilinguals perform semantic judgments. Neuroimage 13, 1155–1163. https://doi.org/10.1006/nimg.2001.0781

Costa, A., Hernández, M., Costa-Faidella, J., Sebastián-Gallés, N., 2009. On the bilingual advantage in conflict processing: now you see it, now you don't. Cognition 113, 135–149. https://doi.org/10.1016/j.cognition.2009.08.001

De Baene, W., Duyck, W., Brass, Mm, Carreiras, M., 2015. Brain Circuit for Cognitive Control is Shared by Task and Language Switching. Journal of Cognitive Neuroscience 27, 1752–65. https://doi.org/10.1162/jocn_a_00817

De Bleser, R., Dupont, P., Postler, J., Bormans, G., Speelman, D., Mortelmans, L., Debrock, M., 2003. The organization of the bilingual lexicon: a PET study, Journal of Neurolinguistics 16, 439–456, https://doi.org/10.1016/S0911-6044(03)00022-8

Fernandez, M., Tartar, J.L., Padron, D., Acosta, J., 2013. Neurophysiological marker of inhibition distinguishes language groups on a non-linguistic executive function test. Brain Cogn. 83, 330–336. https://doi.org/10.1016/j.bandc.2013.09.010

Giezen, M.R., Emmorey, K., 2016. Language co-activation and lexical selection in bimodal bilinguals: Evidence from picture-word interference. Bilingualism 19, 264–276. https://doi.org/10.1017/S1366728915000097

Giroud, N., Baum, S.R., Gilbert, A.C., Phillips, N.A., Gracco, V., 2020. Earlier age of second language learning induces more robust speech encoding in the auditory brainstem in adults, independent of amount of language exposure during early childhood. Brain Lang. 207, 104815. https://doi.org/10.1016/j.bandl.2020.104815

Grant, A.M., Fang, S,-Y., Li, P., 2015. Second language lexical development and cognitive control: A longitudinal fMRI study. Brain and Language 144, 35–47, https://doi.org/10.1016/j.bandl.2015.03.010.

Halsband, U., 2006. Bilingual and multilingual language processing. Journal of Physiology, Paris 2006, 99, 355–369. https://doi.org/10.1016/j.jphysparis.2006.03.016

Hasegawa, M., Carpenter, P.A., Just, M.A., 2002. An fMRI study of bilingual sentence comprehension and workload. Neuroimage 15, 647–660. https://doi.org/10.1006/nimg.2001.1001

Hernandez, A.E., 2009. Language switching in the bilingual brain: What’s next? Brain and Language 109, 133–140. https://doi.org/10.1016/j.bandl.2008.12.005

Hernandez, A.E., Dapretto, M., Mazziotta, J., Bookheimer, S., 2001. Language switching and language representation in Spanish-English bilinguals: An fMRI study. Neuroimage 14, 510–520. https://doi.org/10.1006/nimg.2001.0810

Hernandez, A.E., Martinez, A., Kohnert, K., Figueroa, R., Buffington, R., Juarez, L., Kemper, C., 2000. In Search of the Language Switch: An fMRI Study of Picture Naming in Spanish–English Bilinguals. Brain Lang. 73, 421–431. https://doi.org/10.1006/brln.1999.2278

Jasińska, K.K., Berens, M.S., Kovelman, I., Petitto, L.A., 2017. Bilingualism yields language-specific plasticity in left hemisphere’s circuitry for learning to read in young children. Neuropsychologia 98, 34–45. https://doi.org/10.1016/j.neuropsychologia.2016.11.018

Jeong, H., Sugiura, M., Sassa, Y., Haji, T., Usui, N., Taira, M., Horie, K., Sato, S., Kawashima, R., 2007. Effect of syntactic similarity on cortical activation during second language processing: A comparison of English and Japanese among native Korean trilinguals. Hum. Brain Mapp. 23, 194–204. https://doi.org/10.1002/hbm.20269

Jones, O.P., Green, D.W., Grogan, A., Pliatsikas, C., Filippopolitis, K., Ali, N., Lee, H.L., Ramsden, S., Gazarian, K., Prejawa, S., Seghier, M.L., Price, C.J., 2012. Where, when and why brain activation differs for bilinguals and monolinguals during picture naming and reading aloud. Cereb. Cortex 22, 892–902. https://doi.org/10.1093/cercor/bhr161

Kim, K.H.S., Relkin, N.R., Lee, K.M., Hirsch, J., 1997. Distinct cortical areas associated with native and second languages. Nature 388, 171–174. https://doi.org/10.1038/40623

Kim, S.Y., Qi, T., Feng, X., Ding, G., Liu, L., Cao, F., 2016. How does language distance between L1 and L2 affect the L2 brain network? An fMRI study of Korean-Chinese-English trilinguals. Neuroimage 129, 25–39. https://doi.org/10.1016/j.neuroimage.2015.11.068

Kousaie, S., Chai, X.J., Sander, K.M., Klein, D., 2017. Simultaneous learning of two languages from birth positively impacts intrinsic functional connectivity and cognitive control. Brain Cogn. 117, 49–56. https://doi.org/10.1016/j.bandc.2017.06.003

Kovelman, I., Shalinsky, M.H., Berens, M.S., Petitto, L.-A., 2014. Words in the bilingual brain: an fNIRS brain imaging investigation of lexical processing in sign-speech bimodal bilinguals. Front. Hum. Neurosci. 8, 606. https://doi.org/10.3389/fnhum.2014.00606

Kovelman, I., Baker, S.A., Petitto, L.A., 2008. Bilingual and monolingual brains compared: a functional magnetic resonance imaging investigation of syntactic processing and a possible "neural signature" of bilingualism. Journal of Cognitive Neuroscience 20, 153–169. https://doi.org/10.1162/jocn.2008.20011

Kuzyk, O., Friend, M., Severdija, V., Zesiger, P., Poulin-Dubois, D., 2020. Are there Cognitive Benefits of Code-switching in Bilingual Children? A longitudinal study. Bilingualism: Language and Cognition 23, 542–553. https://doi.org/10.1017/s1366728918001207

Leonard, M.K., Torres, C., Travis, K.E., Brown, T.T., Hagler, D.J., 2011. Language Proficiency Modulates the Recruitment of Non-Classical Language Areas in Bilinguals. PLoS One 6, e18240. https://doi.org/10.1371/journal.pone.0018240

Ma, H., Hu, J., Xi, J., Shen, W., Ge, J., Geng, F., Wu, Y., Guo, J., Yao, D., 2014. Bilingual cognitive control in language switching: an fMRI study of English-Chinese late bilinguals. PLoS One 9:e106468. https://doi.org/10.1371/journal.pone.0106468

Mei, L., Xue, G., Lu, Z.-L., Chen, C., Wei, M., He, Q., Dong, Q., 2015. Long-term experience with Chinese language shapes the fusiform asymmetry of English reading. Neuroimage 110, 3–10. https://doi.org/10.1016/j.neuroimage.2015.01.030

Meschyan, G., Hernandez, A.E., 2006. Impact of language proficiency and orthographic transparency on bilingual word reading: An fMRI investigation. Neuroimage 29, 1135–1140. https://doi.org/10.1016/j.neuroimage.2005.08.055

Michel, M., Kormos, J., Brunfaut, T., Ratajczak, M., 2019. The role of working memory in young second language learners’ written performances. J. Second Lang. Writ. 45, 31–45. https://doi.org/10.1016/j.jslw.2019.03.002

Morgan-Short, K., Steinhauer, K., Sanz, C., Ullman, M.T., 2012. Explicit and implicit second language training differentially affect the achievement of native-like brain activation patterns. J. Cogn. Neurosci. 24, 933–947. https://doi.org/10.1162/jocn_a_00119

Nakamura, K., Kouider, S., Makuuchi, M., Kuroki, C., Hanajima, R., Ugawa, Y., Ogawa, S., 2010. Neural control of cross-language asymmetry in the bilingual brain. Cerebral Cortex 20, 2244–2251. https://doi.org/10.1093/cercor/bhp290

Nicol, A.A.M., De France, K., 2020. Prejudice, Integrativeness, and Motivation to Learn a Second Language: A Mediation Analysis. Psychol. Rep. 123, 420–434. https://doi.org/10.1177/0033294118820509

Olulade, O.A., Jamal, N.I., Koo, D.S., Perfetti, C.A., LaSasso, C., Eden, G.F., 2016. Neuroanatomical evidence in support of the bilingual advantage theory. Cereb. Cortex 26, 3196–3204. https://doi.org/10.1093/cercor/bhv152

Ou, J., Li, W., Yang, Y., Wang, N., Xu, M., 2020. Earlier second language acquisition is associated with greater neural pattern dissimilarity between the first and second languages. Brain Lang. 203, 104740. https://doi.org/10.1016/j.bandl.2019.104740

Perani, D., Paulesu, E., Galles, N.S., Dupoux, E., Dehaene, S., Bettinardi, V., Cappa, S.F., Fazio, F., Mehler, J., 1998. The bilingual brain. Proficiency and age of acquisition of the second language. Brain 121, 1841–1852. https://doi.org/10.1093/brain/121.10.1841

Price, C.J., Green, D.W., Von Studnitz, R., 1999. A functional imaging study of translation and language switching. Brain 122, 2221–2235. https://doi.org/10.1093/brain/122.12.2221

Prior, A., Gollan, T.H., 2011. Good language-switchers are good task-switchers: Evidence from Spanish-English and Mandarin-English bilinguals. J. Int. Neuropsychol. Soc. 17, 682–691. https://doi.org/10.1017/S1355617711000580

Saur, D., Baumgaertner, A., Moehring, A., Büchel, C., Bonnesen, M., Rose, M., Musso, M., Meisel, J.M., 2009. Word order processing in the bilingual brain. Neuropsychologia 47, 158–168. https://doi.org/10.1016/j.neuropsychologia.2008.08.007

Seger, C.A., Prabhakaran, V., Poldrack, R.A., Gabrieli, J.D.E., 2000. Neural activity differs between explicit and implicit learning of artificial grammar strings: An fMRI study. Psychobiology 28, 283–292. https://doi.org/10.3758/BF03331987

Soderfeldt, B., Ingvar, M., Ronnberg, J., Eriksson, L., Serrander, M., Stone-Elander, S., 1997. Signed and spoken language perception studied by positron emission tomography. Neurology 49, 82–87. https://doi.org/10.1212/WNL.49.1.82

Sun, X., Li, L., Ding, G., Wang, R., Li, P., 2019. Effects of language proficiency on cognitive control: Evidence from resting-state functional connectivity. Neuropsychologia 129, 263–275. https://doi.org/10.1016/j.neuropsychologia.2019.03.020

Tu, L., Wang, J., Abutalebi, J., Jiang, B., Pan, X., Li, M., Gao, W., Yang, Y., Liang, B., Lu, Z., Huang, R., 2015. Language exposure induced neuroplasticity in the bilingual brain: a follow-up fMRI study. Cortex 64, 8–19. https://doi.org/10.1016/j.cortex.2014.09.019

Vaughn, K.A., Watlington, E.M., Linares Abrego, P., Tamber-Rosenau, B.J., Hernandez, A.E., 2020. Prefrontal transcranial direct current stimulation (tDCS) has a domain-specific impact on bilingual language control. J. Exp. Psychol. Gen. https://doi.org/10.1037/xge0000956

Verreyt, N., Woumans, E., Vandelanotte, D., Szmalec, A., Duyck, W., 2016. The influence of language-switching experience on the bilingual executive control advantage. Bilingualism 19, 181–190. https://doi.org/10.1017/S1366728914000352

Vingerhoets, G., Van Borsel, J., Tesink, C., van den Noort, M., Deblaere, K., Seurinck, R., Vandemaele, P., Achten, E. 2003. Multilingualism: an fMRI study. NeuroImage 20, 2181–2196. https://doi.org/10.1016/j.neuroimage.2003.07.029

Wang, Y., Xue, G., Chen, C., Xue, F., Dong, Q., 2007. Neural bases of asymmetric language switching in second-language learners: An ER-fMRI study. Neuroimage 35, 862–870. https://doi.org/10.1016/j.neuroimage.2006.09.054

Wartenburger, I., Heekeren, H.R., Abutalebi, J., Cappa, S.F., Villringer, A., Perani, D., 2003. Early setting of grammatical processing in the bilingual brain. Neuron 37, 159–170. https://doi.org/10.1016/S0896-6273(02)01150-9

Williams, J.T., Darcy, I., Newman, S.D., 2016. Modality-specific processing precedes amodal linguistic processing during L2 sign language acquisition: A longitudinal study. Cortex 75, 56–67. https://doi.org/10.1016/j.cortex.2015.11.015

Xi, Y., Liu, L., Hao, G., Abudusadike, Z., Jiang, C., Zhang, J., Wang, B., 2017. Research of cerebral activation in Uygur-speaking and Chinese-speaking participants during verb generation task with functional magnetic resonance imaging. Med. (United States) 96, e7460. https://doi.org/10.1097/MD.0000000000007460

Xu, M., Baldauf, D., Chang, C.Q., Desimone, R., Tan, L.H., 2017. Distinct Distributed patterns of neural activity are associated with two languages in the bilingual brain. Sci. Adv. 3, e1603309. https://doi.org/10.1126/sciadv.1603309

Yetkin, O., Yetkin, F.Z., Haughton, V.M., Cox, R.W., 1996. Use of functional MR to map language in multilingual volunteers. Am. J. Neuroradiol. 17, 473–477.

Zou, L., Abutalebi, J., Zinszer, B., Yan, X., Shu, H., Peng, D., Ding, G., 2012. Second language experience modulates functional brain network for the native language production in bimodal bilinguals. Neuroimage 62, 1367–1375. https://doi.org/10.1016/j.neuroimage.2012.05.062
