# Supplementary material for: General principles governing the amount of neuroanatomical overlap between languages in bilinguals
Source: Neurosci Biobehav Rev. Author manuscript; Available in PMC 2022 Mar 28. (PMC8958881; doi:10.1016/j.neubiorev.2021.08.005)
Supplement: Appendix [file NIHMS1783655-supplement-Appendix.docx]

**APPENDIX 3**

**Tasks applied in single reviewed studies**

Studies with healthy controls:

- sentence listening
- simple declarative sentences
- phoneme categorization
- sentence generation
- retrieval of high and low imaginary words
- visual rhyming judgment
- word repetition
- vowel recognition
- semantic size judgment
- picture selection after story listening
- story watching
- word categorization judgment
- production of syntactic structures
- Ketter String Judgments using artificial grammar,
- Wug test
- word finding vocabulary test
- story reading
- word reading with language switching
- word translation
- backward digit span
- Corsi blocks task, soccer task
- Raven's colored progressive matrices
- auditory go/no-go task, picture naming with distractor words
- vowel recognition, Attentional Control Scale
- action-object switching
- Simon task
- verbal switching
- nonverbal switching
- Cuttell Culture Fair Intelligence Test
- digit naming, lexical decision
- semantic judgment
- silent narration
- task-switching
- Rapid Naming Test
- Kaufman Brief Intelligence Test: Matrices subtest
- letter-number sequencing.

Clinical language mapping studies:

- famous people naming
- translation from L2 to L1
- L1 reading with L2 responding
- synonym generation
- antonym generation
- sentence completion
- alphabet recitation
- verbal instructions
- a semantic judgment of true-false sentences
- color/shape naming
- repetition
